# Autophagy Activation Induces p62-Dependent Autophagic Degradation of Dengue Virus Capsid Protein During Infection

**DOI:** 10.3389/fmicb.2022.889693

**Published:** 2022-07-05

**Authors:** Yaoxing Wu, Tao Zhou, Jiajia Hu, Yishan Liu, Shouheng Jin, Jianfeng Wu, Xiangdong Guan, Jun Cui

**Affiliations:** ^1^Department of Critical Care Medicine, The First Affiliated Hospital of Sun Yat-sen University, Guangzhou, China; ^2^MOE Key Laboratory of Gene Function and Regulation, State Key Laboratory of Biocontrol, School of Life Sciences, Sun Yat-sen University, Guangzhou, China; ^3^State Key Laboratory of Oncology in South China Collaborative Innovation Center for Cancer Medicine, Sun Yat-sen University Cancer Center, Guangzhou, China

**Keywords:** dengue virus, autophagic degradation, p62 (SQSTM1), ubiquitination, cargo receptors, capsid protein (C protein)

## Abstract

In the past decade, dengue virus infection is one of the most prevalent and rapidly spreading arthropod-borne diseases worldwide with about 400 million infections every year. Although it has been reported that the dengue virus could take advantage of autophagy to promote its propagation, the association between selective autophagy and the dengue virus remains largely unclear. Here, we demonstrated that dengue virus capsid protein, the key viral protein for virus assembly, maturation, and replication, underwent autophagic degradation after autophagy activation. Autophagy cargo receptor p62 delivered ubiquitinated capsid protein to autophagosomes for degradation, which could be enhanced by Torin 1 treatments. Further study revealed that the association between p62 and viral capsid protein was dependent on the ubiquitin-binding domain of p62, and the poly-ubiquitin conjugated at lysine 76 of capsid protein served as a recognition signal for autophagy. Consistently, *p62* deficiency in Huh7 cells led to the enhancement of dengue virus replication. Our study revealed that p62 targeted dengue virus capsid protein for autophagic degradation in a ubiquitin-dependent manner, which might uncover the potential roles of p62 in restricting dengue virus replication.

## Introduction

Dengue caused by dengue virus (DENV) is currently one of the most significant and prevalent arthropod-borne viral epidemic diseases with an estimated 400 million infections each year around the world (Bhatt et al., 2013; Byk and Gamarnik, 2016). The incidence of DENV is growing by over 30-fold in the recent decades owing to urbanization, population growth, extensive international travel, and global warming, but clinically specific and effective anti-DENV vaccines and antivirals are still unavailable (Guzman and Harris, 2015; Byk and Gamarnik, 2016; Guzman et al., 2016). Therefore, there is still a pressing need to broaden the acquaintance of the molecular pathogenesis of DENV.

Several recent studies have demonstrated the involvement of autophagy during DENV infection (Deretic and Levine, 2009; Choi et al., 2018). Macroautophagy, hereafter called autophagy, serves as a eukaryotic highly conserved intracellular degradation system, which removes and recycles unwanted or harmful materials in a lysosome-dependent manner (Clarke and Simon, 2019). Autophagy could be highly selective to degrade certain intracellular cargos, including protein aggregates, damaged or superfluous organelles, and invading pathogens via a series of cargo receptors. SQSTM1/p62, NDP52, OPTN, and NBR1, which are well-known cargo receptors containing both ubiquitin-associated (UBA) domain and LC3-interacting regions (LIR), could target certain ubiquitinated substrates to autophagosomes (Dikic and Elazar, 2018; Gatica et al., 2018). Selective autophagy, extensively involved in the host–virus interaction (Viret et al., 2021), directly removes viral particles by targeting viral proteins to autophagosomes for degradation (Kim et al., 2016; Li Y. et al., 2020; Ames et al., 2021; Viret et al., 2021). On the contrary, selective autophagy also degrades antiviral immune factors, such as RIG-I, cGAS, MAVS, TBK1, and IRF3, to avoid excessive interferon (IFN) responses (Chen et al., 2016; Jin et al., 2017; Du et al., 2018; Wu Y. et al., 2021; Xie et al., 2022a). Correspondingly, selective autophagy either restricts or promotes viral replication (Xiao and Cai, 2020; Viret et al., 2021). To date, the association between DENV infection and selective autophagy remains poorly understood, which warrants further investigation.

Dengue virus belongs to *Flavivirus* genus of the *Flaviviridae* family (Byk and Gamarnik, 2016; Guzman et al., 2016). Most flaviviruses are enveloped, icosahedral arboviruses, which contain a positive-strand RNA genome of 9–12 kb. The RNA genome of flaviviruses encodes a polyprotein, which can be further processed into three structural proteins (C, prM, and E) and seven non-structural proteins (NS1, NS2A, NS2B, NS3, NS4A, NS4B, and NS5) (Guzman et al., 2016). As the first translated protein during DENV infection, the C protein plays a crucial role in viral assembly by directly interacting with the viral RNA genome and packaging them for viral particles (Byk and Gamarnik, 2016; Tan et al., 2020). Due to the indispensable role of C protein in the viral assembly, multiple previous studies have tried to illustrate the molecular mechanisms employed by C protein in the formation of viral particles and anchoring prM/E proteins (Byk and Gamarnik, 2016). However, the association between C protein and host factors, especially the host degradation mechanism of C protein, has been seldom mentioned. In this study, we report that host cargo receptor protein p62 selectively targets DENV-2 C protein for autophagic degradation, and this process was enhanced by autophagy activation, such as mTOR inhibitor treatments. C protein undergoes ubiquitination on its lysine 76, which serves as a recognition signal for p62. *p62* deficiency leads to enhanced protein levels of essential C protein and increased susceptibility to DENV infection. Thus, our study reveals the additional role of p62 in restricting the viral replication cycle by directly targeting DENV C protein for autophagic degradation.

## Materials and Methods

### Cell Lines and Culture Conditions

Cell lines HEK293T (human embryonic kidney 293T) and Huh7 (human HCC cell line) were cultured in DMEM medium (Gibco, 10,566,016) with 10% (vol:vol) fetal bovine serum (Gibco, 10,270,160) and 1% glutamine (Gibco, 25,030,081). In order to induce starvation, cells were washed with PBS (Gibco, 10,010,049) and replaced with EBSS (Gibco, 24,010,043). All cells were incubated with 5% CO_2_ in a 37°C incubator.

### Plasmids and siRNA Transfection

Constructs coding for DENV C protein and its mutants were cloned from RNA of DENV strain 16681 into the pcDNA3.1 vector for transient expression and into the FG-EH-DEST (provided by Xiaofeng Qin Laboratory) for retroviral expression. Transfection in HEK293T cells was performed using Lipofectamine 2000 (Invitrogen) according to the manufacturer’s protocol. Chemically synthesized siRNA duplexes were obtained from Sangon Biotech and transfected using Lipofectamine RNAiMAX (Invitrogen) according to the manufacturer’s instructions. RNA oligonucleotides used in this study are as follows:

*p62* siRNA#1: GCAUUGAAGUUGAUAUCGAUTT
*p62* siRNA#2: CUUCCGAAUCUACAUUAAATT

### Generation of Stable Expression and Knockout Cell Lines

The *ATG5*, *BECN1*, *p62*, and *NDP52* knockout (KO) cells were generated as previously described (Jin et al., 2017). In short, target sgRNA sequences were cloned into pLentiCRISPRv2 by cleaving with *Bsm*BI. The lentiviral vectors were co-transfected with an expression plasmid for the vesicular stomatitis virus G protein into the HEK293T cell lines. The virus-containing supernatant was collected 48 h after transfection, and subsequently used to infect cells with polybrene (8 mg/ml). Transduced cells were purified by puromycin selection, and a single clone with sequence verification was selected.

To reintroduce p62 in *p62* KO cells and avoid the recognition of sgRNA, we constructed p62 synonymous mutation in sgRNA-targeted sequences, regarded as sgRNA-resistant mutation, and transfected these mutants in *p62* KO cells.

### Antibodies and Reagents

Horseradish peroxidase (HRP)-anti-Flag (M2) (A8592), anti-β-actin (A1978), and anti-LC3 (ABC929) were purchased from Sigma. Anti-hemagglutinin-HRP (HA) (3F10) and mouse monoclonal anti-c-Myc-HRP (11814150001) were purchased from Roche Applied Science. Anti-dengue capsid (GTX103343) and anti-dengue virus type 2 NS5 antibody (GTX103350) were purchased from Gene Tex. Anti-Beclin-1 (3738) and Anti-ATG5 (12994) were purchased from Cell Signaling Technology. Anti-p62/SQSTM1 (18420-1-AP) and anti-NDP52/CALCOCO2 (12229-1-AP) were purchased from Proteintech Group. Mouse (A7028) and rabbit (A7016) IgG were from Beyotime. Anti-flag M2 affinity beads (A2220) were purchased from Sigma. Protein G agarose (22851) and protein A agarose (22810) were purchased from Pierce.

Puromycin (P9620), rapamycin (37094), Torin 1 (475991), and 3-methyladenine (3-MA) (M9281) were purchased from Sigma. Bafilomycin A1 (S1413) was purchased from Selleck.

### Viruses and Viral Plaque Titration

Dengue virus (strain 16681), which was kindly provided by Dr. Andrew Yueh from National Health Research Institutes in Taiwan, was used in this study. Plaque assays were performed to determine viral titer as previously described (Wu et al., 2017). In short, The DENV-containing supernatants were collected. Vero cells were infected with DENV supernatants for 1 h at room temperature. After washing with PBS, the plate was overlaid with Dulbecco’s modified Eagle’s medium containing 1% low melting point agarose and incubated at 37°C for 72 h before crystal violet staining. Cells were infected at various MOIs Envelope, as previously described (Wu et al., 2017).

### Immunoprecipitation and Immunoblot Analysis

For immunoprecipitation, whole-cell extracts were prepared after infection, transfection, or stimulation with indicated ligands, followed by incubation overnight with anti-Flag agarose gels (Sigma) or appropriate antibody with protein A/G agarose gels (Pierce) at 4°C. Beads were subsequently washed 3–5 times using low-salt lysis buffer (50 mM HEPES, 150 mM NaCl, 1 mM EDTA, 10% glycerol, 1.5 mM MgCl_2_, and 1% Triton X-100), and immunoprecipitates were eluted with × 2 SDS loading buffer and resolved by SDS-PAGE. Then proteins were transferred to PVDF membranes (Bio-Rad) and further incubated with the appropriate antibodies. Immobilon Western Chemiluminescent HRP Substrate (Millipore, WBKLS0500) was used for protein detection.

### Quantitative RT-PCR

The total RNA was extracted from cells using the Trizol reagent (Invitrogen) according to the manufacturer’s instructions. For RT-PCR analysis, cDNA was generated with HiScript^®^ II Q RT SuperMix for qPCR (+ gDNA wiper) (Vazyme, R223-01) and was analyzed by quantitative real-time PCR using the × 2 RealStar Green Power Mixture (GenStar). All data were normalized to *RPL13A* expression. Primer sequences are listed below:

*RPL13A* Forward: 5′-GCCATCGTGGCTAAACAGGTA-3′
Reverse: 5′-GTTGGTGTTCATCCGCTTGC-3′
*DENV-2 Envelope* Forward: 5′-TGCCCAACACAAGGRG AACC -3′
Reverse: 5′-GCRCAGGTCACAATGCCYCC-3′
*DENV-2 NS5* Forward: 5′-ACAAGTCGAACAACCTGG TCCAT-3′
Reverse: 5′-GCCGCACCATTGGTCTTCTC-3′
*DENV-2 Capsid* Forward: 5′-ACGCGAGAGAAACCGC GTGTCGACTGT-3′
Reverse: 5′-AAACGAAGGAACGCCACCAGGGCCA-3′

### Fluorescence Microscopy

Cells were cultured on glass-bottom culture dishes (Nest Scientific). After stimulation, cells were fixed with 4% paraformaldehyde for 10 min and washed with cold PBS for three times before permeabilizing with 0.1%Triton X-100 diluted in PBS for 10 min at room temperature. After washing with cold PBS for three times, cells were blocked with 6% goat serum (Boster Biological, AR1009) for 1 h at room temperature, and then incubated with primary antibodies diluted in 6% goat serum overnight. Cells were washed with PBS for three times and subsequently incubated with fluorescently labeled secondary antibodies (Alexa Fluor 488- and Alexa Fluor 568-conjugated antibodies against mouse and rabbit) for 1 h. Confocal images were captured by microscope (TCS SP8 STED 3X, Leica) equipped with × 100 1.40 NA oil objectives. The images were processed for gamma adjustments using Leica AS Lite or ImageJ software (National Institutes of Health).

The co-localization analysis of C protein with the LC3 or p62 was estimated through Pearson’s correlation coefficients (R) using the PSC co-localization plug-in (ImageJ, NIH). The R values range between −1 (perfect negative correlation) and + 1 (perfect positive correlation), with 0 corresponding to no correlation (French et al., 2008).

### Statistical Analyses

Images were analyzed with ImageJ. All data are presented as mean ± SEM unless from independent determinations, and statistical analyzes were done using the software Graphpad Prism (GraphPad Software, Inc, La Jolla, CA, United States). Differences in means were tested for statistical significance with an unpaired two-tailed Student’s *t*-test. *p < 0.05; ^**^p < 0.01; ns, not significant.

## Results

### Autophagy Reduces the Abundance of Dengue Virus (DENV) Capsid Protein

**FIGURE 1:**
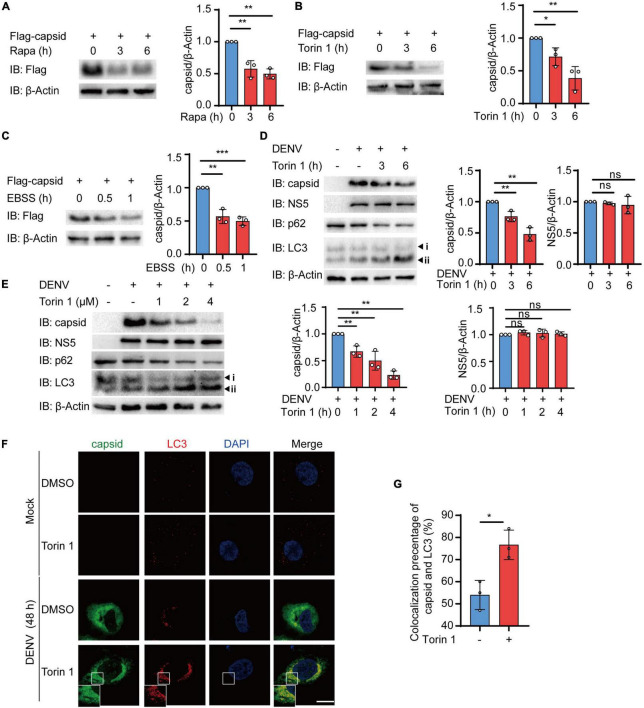
Autophagy reduces the protein abundance of dengue virus (DENV) capsid protein. **(A–C)** 293T cells that stably expressed C protein were treated with 4 μM Rapa **(A)**, 2 μM Torin 1 **(B)**, or earle’s balanced salt solution (EBSS) **(C)** for indicated time points. The cell lysates were subjected to immunoblot analysis, and the quantified levels of C protein were normalized to β-actin. **(D)** Huh7 cells were infected with DENV (MOI = 0.5), followed by 2 μM Torin 1 treatments for indicated time points before harvesting. The cell lysates were immunoblotted with indicated antibodies, and the quantified levels of C protein were normalized to β-actin. **(E)** Huh7 cells were infected with DENV (MOI = 0.5), followed by indicated doses of Torin 1 treatments for 6 h before harvesting. The cell lysates were immunoblotted with indicated antibodies, and the quantified levels of C protein (left) or NS5 (right) were normalized to β-Actin. **(F)** Confocal microscopy of Huh7 cells infected with DENV (MOI = 0.5) for 24 h and treated with Torin 1 (2 μM) for 6 h, followed by labeling of C protein and LC3 with specific primary antibody and a CF568 goat anti-mouse IgG secondary antibody (red) and Alexa Fluor 488 conjugated anti-rabbit-IgG secondary antibody (green). Scale bar, 10 μm. **(G)** Pearson’s correlation coefficients of DENV C protein and LC3 in **(F)**. Data represent replicate measurements in at least 10 cells for one independent experiment, and these experiments have been replicated three times. Data in **(A–E,G)** are expressed as mean ± SEM of three independent experiments. *p < 0.05, **p < 0.01, NS, not significant (two-tailed Student’s *t*-test). All the experiments are representatives of three independent biological experiments with similar results.

The accumulating evidence has indicated the close relationship between autophagy and DENV during infection, while several DENV components have been previously proved to be co-localized with autophagosomes (Heaton and Randall, 2010; Zhang et al., 2018; Li M. Y. et al., 2020; Lu et al., 2020). Due to the critical role of DENV C protein in virion assembly and viral replication, we first investigated whether DENV C protein underwent degradation after autophagic induction. To detect the protein level of DENV C protein after autophagic activation, we stably expressed the DENV C protein in 293T cells using lentivirus as previously described (Jin et al., 2017). Subsequently, we checked the level of DENV C protein in the presence of autophagy inducers, such as rapamycin (Rapa) and Torin 1, two mTOR inhibitors. Immunoblot results showed that both Rapa and Torin 1 treatments led to the reduction of C protein levels (Figures 1A,B). To confirm these results, we also constructed C protein in pcDNA3.1 plasmid with GFP tag and transfected C protein in 293T cells, followed by Rapa or Torin 1 treatments. Similarly, treatments of autophagic activators also reduced the protein level of C protein when it was transiently expressed, compared with GFP control (Supplementary Figures 1A,B). We next checked the C protein level upon starvation-induced autophagy activation under Earle’s balanced salt solution (EBSS) condition. Consistently, EBSS-stimulated autophagy could also reduce C protein levels (Figure 1C and Supplementary Figure 1C). To further investigate the role of autophagic inducers on DENV C protein, we treated the DENV-infected Huh7 cells with Torin 1 in different doses and different time courses. Analogously, Torin 1 stimulation resulted in the reduction of C protein in DENV-infected cells in both time-course and dose-dependent manner (Figures 1D,E). Correspondingly, treatments of Torin 1 did not significantly affect the protein level of DENV NS5 (Figures 1D,E). To confirm the autophagic reduction of C protein in DENV-infected cells, we stained the DENV-infected Huh7 cells with a C protein antibody. Fluorescence images revealed that the percentage of C-positive cells decreased from 36 to 20% on average and relative GFP intensity was also reduced after Torin 1 treatment (Supplementary Figures 1D,F). Furthermore, C protein displayed an autophagosome distribution in DENV-infected cells, and Torin 1 treatments led to the enhancement of the co-localization between C protein and LC3 (Figures 1F,G). To further investigate the correlation between the autophagy-induced reduction of C protein and DENV replication, we detected DENV C protein level as well as DENV replication in a time-dependent manner. Immunoblot analysis showed that C protein level was decreased after Torin 1 treatments for 6 h, however; DENV replication level was reduced only after Torin 1 treatments for 24 h, indicating the out-of-sync effect of autophagy in C protein reduction and DENV replication (Supplementary Figures 1G,I). Together, these results revealed that autophagy activation decreased the protein abundance of DENV C protein, which suggested the involvement of autophagy in regulating DENV C protein.

### Dengue Virus (DENV) Capsid Protein Is Degraded in an Autophagy-Dependent Manner

**FIGURE 2:**
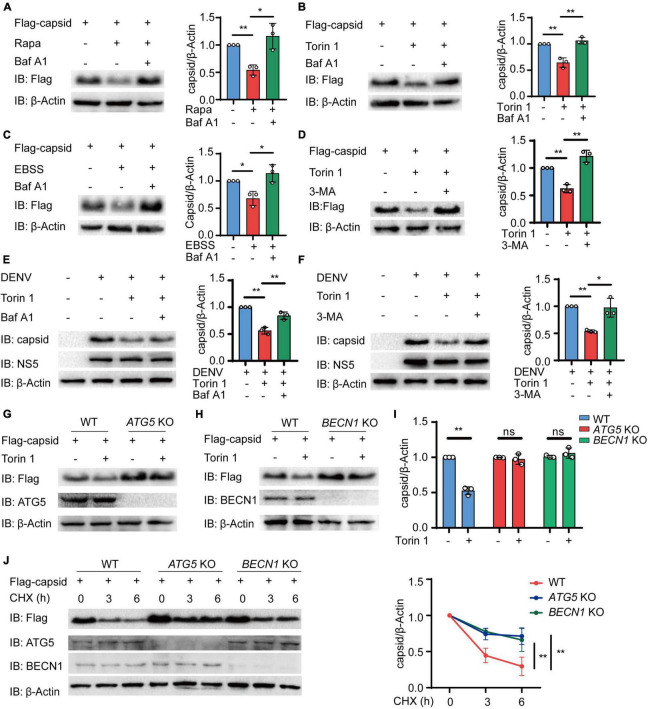
Dengue virus (DENV) capsid protein is degraded in an autophagy-dependent manner. **(A–C)** 293T cells that stably expressed C protein were pre-treated with 0.2 μM Baf A1 for 6 h, followed by treatments with 4 μM Rapa **(A)** and 2 μM Torin 1 **(B)** for 6 h or earle’s balanced salt solution (EBSS) **(C)** for 1 h. The cell lysates were immunoblotted with indicated antibodies, and the quantified levels of C protein were normalized to β-actin. **(D)** 293T cells that stably expressed C protein were pre-treated with 10 mM 3-MA for 6 h, followed by 2 μM Torin 1 treatments for 6 h before harvesting. The cell lysates were immunoblotted with indicated antibodies, and the quantified levels of C protein were normalized to β-actin. **(E,F)** DENV (MOI = 0.5)-infected Huh7 cells were treated with 0.2 μM Baf A1 for 6 h **(E)** or 10 mM 3-MA **(F)** for 6 h, followed by 2 μM Torin 1 treatments for 6 h before harvesting. The cell lysates were immunoblotted with indicated antibodies, and the quantified levels of C protein were normalized to β-actin. **(G,H)** Flag-C protein was transfected into WT, *ATG5* KO **(G)**, or *BECN1* KO **(H)** cells, followed by 2 μM Torin 1 treatment for 6 h. The cell lysates were immunoblotted with indicated antibodies. **(I)** Quantified levels of C protein from **(G,H)** were normalized to β-actin. **(J)** Flag-C protein was transfected into WT, *ATG5* KO, or *BECN1* KO cells, followed by treatments with cycloheximide (CHX) (100 ng/ml) for indicated time points. The cell lysates were subjected to immunoblot analysis with the indicated antibodies, and the quantified levels of C protein were normalized to β-actin. Data in **(A–F,I,J)** are expressed as mean ± SEM of three independent experiments. *p < 0.05, **p < 0.01, ns, not significant (two-tailed Student’s *t*-test). All the experiments are representatives of three independent biological experiments with similar results.

We next sought to determine how autophagy reduces the protein level of C protein. To detect whether C protein undergoes autophagic degradation, we investigated the reduction of C protein upon inhibition of lysosomal activity with bafilomycin A1 (Baf A1). Immunoblot results showed that the reduction of C protein, which was induced by different autophagic inducers including Rapa, Torin 1, and EBSS, could be abrogated by Baf A1 (Figures 2A–C and Supplementary Figures 2A–C). In addition to lysosome inhibitor, 3-methyladenine (3-MA), the widely used autophagic inhibitor via inhibiting class III PI3K, could also block the reduction of C protein abundance induced by Torin 1 (Figure 2D). Consistently, Baf A1 and 3-MA treatments could inhibit the decrease of C protein in DENV-infected cells (Figures 2E,F). We next determined C protein turnover rates in the presence of cycloheximide (CHX) in DENV-infected cells after Baf A1 and 3-MA treatments. CHX chase assay showed that both Baf A1 and 3-MA treatments could slower C protein degradation rate in DENV-infected cells (Supplementary Figures 2D,F). To further verify whether C protein underwent autophagy degradation after autophagy induction, we detected the protein level of C protein in *ATG5* and *BECN1* KO cells as previously described (Jin et al., 2017), in which autophagy was severely restricted. Similarly, Torin 1- or EBSS-induced reduction of C protein was impaired in *ATG5* and *BECN1* KO cells (Figures 2G–I and Supplementary Figures 2G,H). In addition, the CHX chase assay revealed that turnover rates of C protein were also weakened in *ATG5* and *BECN1* KO cells (Figure 2J). Collectively, these results indicated that DENV capsid protein was degraded in an autophagy-dependent manner.

### Autophagy Induction Enhances the Interaction Between p62 and Dengue Virus (DENV) Capsid Protein

**FIGURE 3:**
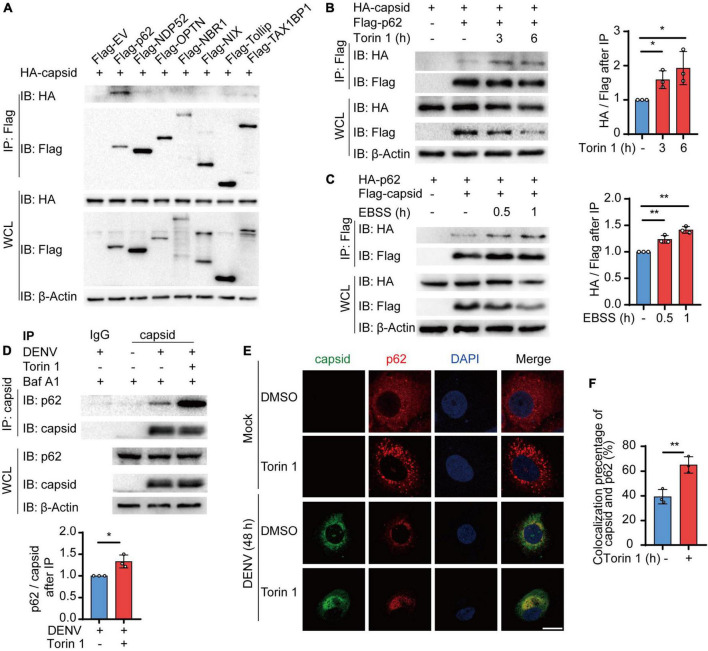
Cargo receptor p62 interacts with dengue virus (DENV) capsid protein during viral infection. **(A)** 293T cells were transfected with plasmids encoding HA-C protein and indicated Flag-tagged cargo receptors, followed by immunoprecipitation (IP) with anti-Flag beads and immunoblot analysis with anti-HA. **(B)** Coimmunoprecipitation and immunoblot analysis of 293T cells transfected with plasmids expressing HA-C protein and Flag-p62 with 2 μM Torin 1 treatments for indicated time points. The ratios between HA and Flag after immunoprecipitation were analyzed. **(C)** Coimmunoprecipitation and immunoblot analysis of 293T cells transfected with plasmids expressing HA-p62 and Flag-C protein with earle’s balanced salt solution (EBSS) treatments for indicated time points. The ratios between HA and Flag after immunoprecipitation were analyzed. **(D)** Coimmunoprecipitation and immunoblot analysis of Huh7 cells infected with DENV for 48 h followed by Torin 1 and Baf A1 treatments. The ratios between p62 and C protein after immunoprecipitation were analyzed. **(E)** Confocal microscopy of Huh7 cells infected with DENV (MOI = 0.5) for 24 h and treated with Torin 1 (2 μM) for 6 h, followed by labeling of C protein and p62 with specific primary antibody and a CF568 goat anti-mouse IgG secondary antibody (red) and Alexa Fluor 488 conjugated anti-rabbit-IgG secondary antibody (green). Scale bar, 10 μm. **(F)** Pearson’s correlation coefficients of DENV C protein and p62 in **(E)**. Data represent replicate measurements in at least 10 cells from three independent experiments. Data in **(A–D,F)** are expressed as mean ± SEM of three independent experiments. *p < 0.05, **p < 0.01 (two-tailed Student’s *t*-test). All the experiments are representatives of three independent biological experiments with similar results.

The accumulating evidence suggested that autophagy cargo receptors play a central role in delivering viral components to the autophagosomes for selective degradation (Viret et al., 2021). Subsequently, we determined which cargo receptors control the autophagic degradation of C protein. Coimmunoprecipitation assay revealed that C protein mainly interacted with p62, NDP52, and TAX1BP1, and p62 exhibited the stronger interaction with C protein among these receptors (Figure 3A). Hence, we focused on p62 and investigated the relationship between p62 and C protein. Moreover, we found that both Torin 1 and EBSS treatments enhanced the association between C protein and p62 when we co-transfected C protein and p62 in 293T cells (Figures 3B,C). Consistently, endogenous p62 was also shown to interact with DENV C protein in DENV-infected cells, while Torin 1 treatments promoted their interaction (Figure 3D). The immunofluorescence analysis indicated that DENV C protein co-localized with endogenous p62 during DENV infection, while Torin 1 treatments further enhanced the co-localization between C protein and p62 (Figures 3E,F). Together, our results suggested that p62 interacted with DENV C protein during infection, while autophagic inducer could further facilitate their associations.

### Cargo Receptor p62 Mediates the Autophagic Degradation of Dengue Virus (DENV) Capsid Protein

**FIGURE 4:**
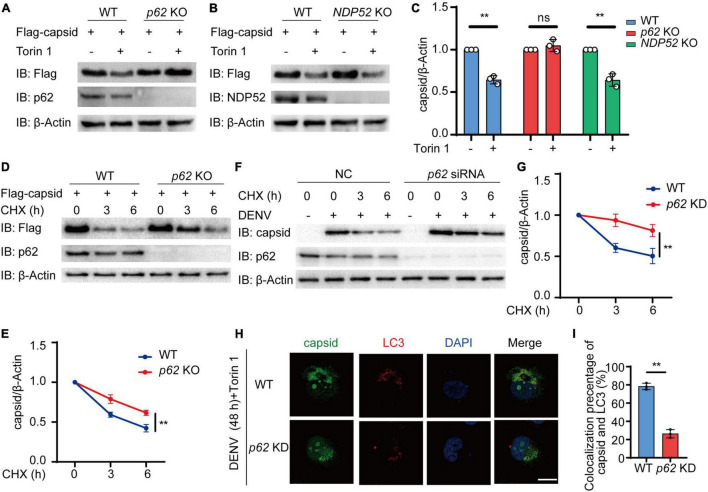
p62 mediates the autophagic degradation of dengue virus (DENV) capsid protein. **(A,B)** Flag-C protein was transfected into WT, *p62* KO **(A),** or *NDP52* KO **(B)** cells, followed by 2 μM Torin 1 treatments for 6 h. The cell lysates were immunoblotted with indicated antibodies. **(C)** The quantified levels of C protein from **(A,B)** were normalized to β-actin. **(D)** Flag-C protein was transfected into WT or *p62* KO cells, followed by CHX (100 ng/ml) treatments for indicated time points. The cell lysates were subjected to immunoblot analysis with the indicated antibodies. **(E)** The quantified levels of C protein in **(D)** were normalized to β-actin. **(F)** Huh7 cells transfected with control (NC) or *p62*-specific siRNA were infected with DENV (MOI = 0.5) for 48 h, followed by CHX (100 ng/ml) treatments for indicated time points. The cell lysates were immunoblotted with indicated antibodies. **(G)** The quantifiedlevels of C protein in **(F)** were normalized to β-actin. **(H)** Confocal microscopy of WT or *p62* KD Huh7 cells infected with DENV (MOI = 0.5) for 24 h and Torin 1 (2 μM) treatments for 6 h before harvesting, followed by labeling of C protein and LC3 with specific primary antibody and a CF568 goat anti-mouse IgG secondary antibody (red) and Alexa Fluor 488 conjugated anti-rabbit-IgG secondary antibody (green). Scale bar, 10 μm. **(I)** Pearson’s correlation coefficients of DENV C protein and LC3 in **(H)**. Data represent replicate measurements in at least 10 cells from three independent experiments. Data in **(C,E,G,I)** are expressed as mean ± SEM of three independent experiments. **p < 0.01, ns, not significant (two-tailed Student’s *t*-test). All the experiments are representatives of three independent biological experiments with similar results.

We next determined whether cargo receptor p62 was involved in the autophagic degradation of C protein. We investigated Torin 1-induced autophagic degradation of C protein in the absence of p62 or NDP52, another cargo receptor closely related to antiviral immunity. We observed that Torin 1-induced degradation of C protein was inhibited in *p62* KO cells, while C protein could still undergo autophagic degradation in *NDP52* KO cells (Figures 4A–C). Additionally, CHX-induced turnover of C protein was decreased in *p62* KO cells (Figures 4D,E). To confirm whether p62 affects the C protein turnover, we next transfected *p62* siRNA followed by DENV infection, and detected C protein turnover in DENV-infected cells. CHX chase assay revealed that the C protein degradation rate was reduced in *p62* knockdown (KD) cells (Figures 4F,G). Immunofluorescence results showed that overlap between C protein and endogenous LC3 was abrogated in *p62* KD cells (Figures 4H,I). Altogether, our results suggested that p62 modulated the autophagic degradation of C protein.

### Ubiquitination on Dengue Virus (DENV) Capsid at Lysine 76 Is Essential for Its Autophagic Degradation *via* p62

**FIGURE 5:**
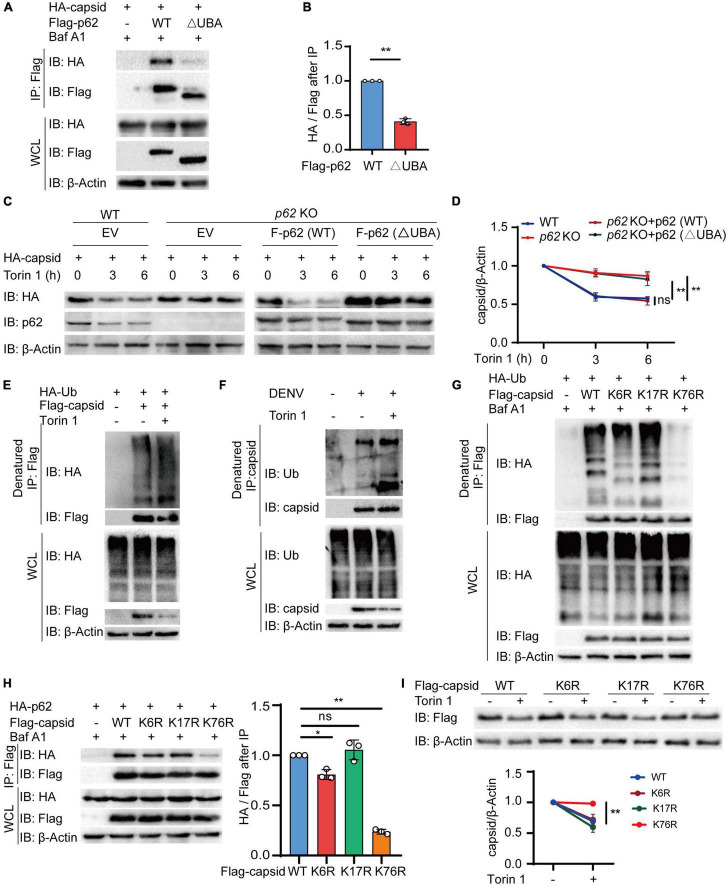
p62 recognizes dengue virus (DENV) capsid protein through its ubiquitination at lysine 76. **(A)** Coimmunoprecipitation and immunoblot analysis of 293T cells transfected with plasmids expressing HA-p62 and Flag-C protein and its △UBA mutant with 0.2 μM Baf A1 treatments for 6 h. **(B)** The ratios between HA and Flag after immunoprecipitation from **(A)** were analyzed. **(C)** Flag-p62 protein or its △UBA mutant was transfected into WT or *p62* KO 293T cells, followed by HA-C transfection for 24 h and 2 μM Torin 1 treatments for indicated time points before harvesting. The cell lysates were immunoblotted with indicated antibodies. **(D)** The quantified levels of C protein from **(C)** were normalized to β-actin. **(E)** Denatured immunoprecipitation and immunoblot analysis of 293T cells transfected with plasmids expressing Flag-C protein and HA-ubiquitin with or without 2 μM Torin 1 treatments for 6 h. **(F)** Denatured immunoprecipitation and immunoblot analysis of Huh7 cells infected with DENV (MOI = 0.5) for 48 h, followed with or without 2 μM Torin 1 treatments for 6 h. **(G)** Denatured immunoprecipitation and immunoblot analysis of 293T cells transfected with plasmids expressing Flag-C protein (WT, K6R, K17R, or K76R) and HA-ubiquitin with 0.2 μM Baf A1 treatments for 6 h. **(H)** Coimmunoprecipitation and immunoblot analysis of 293T cells transfected with plasmids expressing Flag-C protein (WT, K6R, K17R, or K76R) and HA-p62 with 0.2 μM Baf A1 treatments for 6 h. **(I)** Flag-C protein or its mutants (K6R, K17R, or K76R) were transfected into 293T cells, followed by 2 μM Torin 1 treatments for 6 h. The cell lysates were immunoblotted with indicated antibodies and quantified levels of C protein were normalized to β-actin. Data in **(B,D,H,I)** are expressed as mean ± SEM of three independent experiments. *p < 0.05, **p < 0.01, ns, not significant (two-tailed Student’s *t*-test). All the experiments are representatives of three independent biological experiments with similar results.

Ubiquitin chains on the substrates are regarded as a major recognition signal for cargo receptors (Komander and Rape, 2012; Deretic et al., 2013). To recognize ubiquitin chains, cargo receptors always harbor a UBA domain. To detect whether the UBA domain of p62 is necessary for autophagic degradation of C protein, we constructed the UBA deletion (△UBA) mutant of p62 to determine its association with C protein. Coimmunoprecipitation results revealed that △UBA mutant of p62 largely impaired its interaction with C protein, compared with wild-type (WT) p62 (Figures 5A,B). To verify the roles of the UBA domain of p62, we next reconstituted sgRNA-resistant WT or △UBA mutant of p62 in *p62* KO cells to detect the autophagic degradation of C protein, and found that the △UBA mutant of p62 reduced the Torin 1-induced degradation of C protein, suggesting UBA domain was required for the autophagic degradation of C protein (Figures 5C,D). Consistently, the CHX chase assay also revealed that the turnover rates of C protein were reduced in *p62* KO cells or △UBA mutant-reconstituted cells (Supplementary Figures 3A,B). Collectively, these results suggested that the UBA domain of p62 was involved in the autophagic degradation of C protein.

As the UBA domain of p62 is responsible for recognizing the ubiquitinated substrates, it is reasonable to assume that ubiquitin chains conjugated on C protein act as a recognition signal for p62. To verify this assumption, we next checked the ubiquitination of C protein and found that C protein was capable of undergoing ubiquitination (Supplementary Figure 3C). Furthermore, autophagy activation with Torin 1 treatments enhanced the ubiquitination level of C protein (Figure 5E). In addition, we also observed the elevated endogenous ubiquitination of C protein after Torin 1 treatments in DENV-infected cells (Figure 5F). To investigate the ubiquitin-binding sites of C protein, we employed an *in silico* method UbPred (Radivojac et al., 2010) and generated three C protein mutants containing a single substitution [from lysine (K) to arginine (R)] in the top three predicted ubiquitination sites, thus generating C*^K6R^*, C*^K17R^*, and C*^K76R^* mutants. Among these mutants, the C*^K76R^* mutant exhibited reduced ubiquitination capacity (Figure 5G and Supplementary Figure 3D). Furthermore, our results showed that the interaction between C protein and p62 was remarkedly decreased when K76 of C protein was mutated, compared with WT or other mutants of C protein (Figure 5H). Torin 1-induced autophagic degradation of C*^K76R^* mutant was also disrupted compared to WT or other mutants of C protein (Figure 5I). In addition, the CHX chase assay showed that the turnover rates of C^*K76R*^ mutant were also slowed down (Supplementary Figures 3E,F). Taken together, these results suggested that K76 in DENV C protein is an essential residue for its ubiquitination, which served as a recognition signal for p62.

### p62 Restricts the Replication of Dengue Virus (DENV)

**FIGURE 6:**
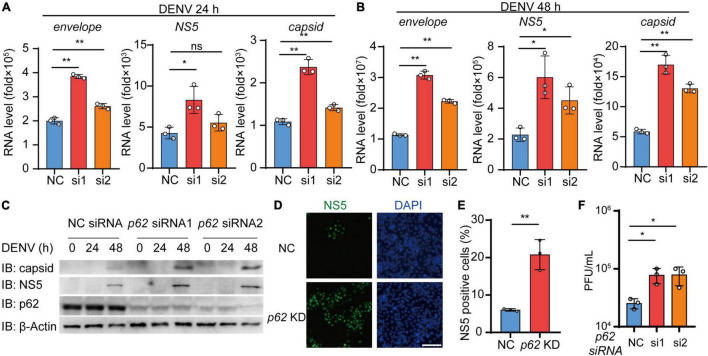
p62 restricts the replication of dengue virus (DENV). **(A,B)** Huh7 cells transfected with control (NC) or *p62*-specific siRNA were infected with DENV (MOI = 0.5) for 24 h **(A)** and 48 h **(B)**. Relative RNA levels of DENV *E protein*, *C protein*, and *NS5* were measured by real-time PCR. **(C)** Huh7 cells were transfected with NC or *p62*-specific siRNA, followed by infection with DENV (MOI = 0.5) at different time points, and the lysates were immunoblotted with indicated antibodies. **(D)** Confocal microscopy of WT or *p62* KD Huh7 cells infected with DENV (MOI = 0.5) for 24 h, followed by labeling of NS5 with a specific primary antibody and an Alexa Fluor 488 conjugated anti-rabbit-IgG secondary antibody (green). Scale bar, 200 μm. **(E)** The graphs display the relative percentage of positive cells in **(D)**. Data represent replicated measurements in at least 1,000 cells for one independent replicate. **(F)** Viral plaque analyzes of WT or *p62* KD Huh7 cells infected with DENV (MOI = 0.5) for 48 h. Data in **(A,B,E,F)** are expressed as mean ± SEM of three independent experiments. *p < 0.05, **p < 0.01, ns, not significant (two-tailed Student’s *t*-test). All the experiments are representatives of three independent biological experiments with similar results.

Given that p62 controls the autophagic degradation of DENV C protein, we next examined the involvement of p62 during DENV infection. We transfected Huh7 cells using siRNA targeting p62 followed by DENV infection and performed real-time PCR analysis to confirm the replication level of DENV. The results showed that the replication of the DENV genome was increased in *p62* KD cells relative to control cells (Figures 6A,B and Supplementary Figure 4A). Similarly, the immunoblot assay showed that the protein levels of C protein and NS5 were increased in *p62* KD cells (Figure 6C). By staining DENV NS5 protein, we found that DENV spread from 7% to 21% of cells on average in *p62* KD cells compared with control cells (Figures 6D,E). To further support the antiviral role of p62, we performed a viral plaque assay to determine DENV titers in *p62* KD cells. Consistently, the production of infectious viruses was also enhanced in *p62* KD cells (Figure 6F). To determine the restriction role of p62 on DENV infection, we detected the protein abundance during DENV infection and observed only a high titer (MOI = 5) of DENV could lead to the reduction of p62 (Supplementary Figure 4B). Overall, these results indicated that p62 restricted the spread and replication of DENV, suggesting that p62 could serve as an anti-DENV factor by promoting the autophagic degradation of DENV C protein.

## Discussion

The repertoire of selective autophagy functions during viral infection has been greatly expanded in recent decades (Choi et al., 2018; Viret et al., 2021). The most intrinsic immune function of selective autophagy is its role in the direct removal of intracellular viral constituents or virions (Viret et al., 2021). Several well-known cargo receptors are reported to participate in the autophagic degradation of viral components. For instance, p62 could target sindbis virus (SINV) capsid protein to autophagosomes for degradation and restrict SINV replication (Orvedahl et al., 2010). Similarly, capsid proteins of chikungunya alphavirus (CHIKV), avibirnavirus, and picornavirus foot-and-mouth disease virus (FMDV) could also be the targets for p62-mediated autophagic degradation (Berryman et al., 2012; Judith et al., 2013; Li Y. et al., 2020). Other famous autophagy receptors also participate in cytoprotection by delivering viral components to autophagosomes for degradation. NDP52 could induce autophagic degradation of CHIKV nsp2 (Judith et al., 2013). OPTN could target herpes simplex virus-1 (HSV-1) essential protein VP16 and gB, thus reducing the replication of HSV-1 and hence herpes encephalitis (Ames et al., 2021). Interestingly, several host antiviral factors, including TRIM5α, SMURF1, FAM134B, and SCOTIN, exhibit their potential to transport viral elements to autophagosomes as cargo receptors (Orvedahl et al., 2011; Kim et al., 2016; Ribeiro et al., 2016; Lennemann and Coyne, 2017). In addition to directly mediating the autophagic degradation of viral constituents, selective autophagy receptors could also target host molecules hijacked by viruses. p62 is shown to promote the autophagic degradation of Tat, a host factor kidnaped by HIV for viral transcription, and impair the HIV replication (Sagnier et al., 2015). Previous studies have outlined the close relationship between DENV infection and autophagy. It has been reported that DENV could utilize host autophagy, especially lipophagy, to increase energy production for viral replication, while overexpression of p62 is also shown to limit the replication of DENV (Metz et al., 2015; Zhang et al., 2018). The association between selective autophagy and DENV infection remains controversial, and the detailed mechanisms involved in the autophagic degradation of DENV constituents remain unclear. Our study reveals that p62 could also contribute to anti-DENV intrinsic cellular defense during infection. Mechanistically, p62 could sense DENV C protein and deliver C protein to autophagosomes for degradation, thus restricting the DENV replication. Thus, our results indicate a prominent antiviral role for p62 as well as selective autophagy in the life cycle of DENV, which might suggest novel targets and potential strategies for developing rational anti-DNEV therapy.

As a bridge to connect cargos and autophagosomes, autophagic cargo receptors are responsible for sensing the recognition signal from targeted substrates and delivering them to autophagosomes through LC3 (Dikic and Elazar, 2018; Gatica et al., 2018). Ubiquitination is the most common recognition signal for cargo receptors (Komander and Rape, 2012). During viral infection, viral components with ubiquitin chains might also serve as a recognition signal for selective autophagy (Viret et al., 2021). It has been reported that DENV C protein could undergo ubiquitination during viral infection. The ubiquitination of C protein at its N-terminal end is required for the uncoating of the DENV genome (Byk et al., 2016). Ubiquitination of C protein also mediates the degradation of DENV C protein through the ubiquitin–proteasome system (Byk et al., 2016). In the current study, we demonstrate that DENV C protein undergoes ubiquitination on its lysine residue 76, and the ubiquitination of C protein could also serve as a recognition signal for autophagic receptor p62. Treatment with autophagic activators, such as Torin 1, could not only enhance the ubiquitination level of DENV C protein, but also promote lipid phosphatidylethanolamine of LC3, which is necessary for autophagosome formation (Lamark et al., 2017). Therefore, Torin 1 could enhance p62 recognition of C protein and allow C protein to localize on autophagosomes. In addition, K76R mutation of C protein, which hinders the ubiquitination of C protein, leads to attenuated interaction between p62 and C protein, as well as the p62-mediated degradation of C protein. Thus, it can be inferred that lysine residue 76 of DENV C protein is covalently modified by ubiquitin chains during infection. Subsequently, p62 targets ubiquitinated C protein and serves as a bridge to connect DENV C protein and autophagosomes, thus leading to the selective autophagic degradation of C protein and restriction of DENV replication.

In addition to eliminating the invading pathogen, p62 also functions as a signaling hub during viral infection. Through interacting with TRAF6 via its TRAF6-binding motif, p62 is able to mediate TRAF6 oligomerization and subsequent NF-κB activation by enhancing the K63-linked polyubiquitination of TRAF6 (Moscat and Diaz-Meco, 2009; Sumpter and Levine, 2011). As most studies reveal that p62 accumulation facilitates the activation of NF-κB signaling, therefore, by extrapolation, p62 is also involved in pro-inflammatory NF-κB activation during viral infection (Moscat and Diaz-Meco, 2009; Sumpter and Levine, 2011). Additionally, enhanced p62 aggregates also lead to increased death in the viral-infected cell, as p62 participates in caspase-8-mediated extrinsic apoptosis signaling through interacting with caspase-8 (Jin et al., 2009). In addition to the effect of p62 on NF-κB pro-inflammatory signaling, enforced expression of p62 also leads to the enhancement of ROS generation, which contributes to elevated IFN and inflammasome activation (Saitoh et al., 2008; Tal et al., 2009). On the contrary, p62 can also reduce excessive inflammatory responses through selective autophagy (Liu et al., 2022). p62 is shown to restrict NF-κB inflammatory signaling by mediating selective autophagic degradation of IKKβ and p65 (Feng et al., 2017; Liu et al., 2018). Moreover, p62 could also reduce inflammasome activation by removing damaged mitochondria or inflammasome component AIM2 through selective autophagy (Liu et al., 2016; Zhong et al., 2016). Similar to the effect in restricting inflammatory, p62 could also disrupt viral-triggered type I IFN immune responses. p62 has been reported to control IFN antiviral responses by promoting autophagic degradation of several key factors in the IFN pathway, including RIG-I, cGAS, MAVS, and IRF3 during viral infection (Xian et al., 2020; Liu et al., 2021; Xie et al., 2022b). Thus, the whole performance of p62 during DENV infection has to be pondered in light of the fact that p62 functions as a signaling hub to coordinate autophagy, viral elimination, IFN antiviral responses, and inflammation.

Due to co-evolution between DENV and their hosts, DENV seems to develop diverse strategies to minimize host restriction on virus replication. A previous study from Metz’s team revealed that overexpression of p62 could suppress DENV replication (Metz et al., 2015). Consistently, our current study showed that *p62* knockdown in Huh7 cells could enhance the replication of DENV. Additionally, p62 is shown to target DENV C protein for autophagic degradation, suggesting the capacity of p62 in counteracting DENV. However, DENV seems to evolve several strategies to lower p62 restrictions on DENV infection. Straightforwardly, DENV is reported to directly degrade p62 protein during infection (Metz et al., 2015). As we showed in Supplementary Figure 4B, DENV could reduce the level of p62 protein with a high virus titer at the late virus infection stage, which indicated that DENV might restrict the role of p62 in a dose-dependent and time-course manner. In addition to direct degradation of p62, DENV is also shown to inhibit autophagic flux at the later stage of infection (Metz et al., 2015). By regulating autophagic flux, DENV may reduce autophagic degradation of DENV particles at a late infection stage. In the current manuscript, DENV C protein fails to exhibit a significant decrease during infection without autophagy activation, such as starvation or mTOR inhibitor. A previous study also showed that lysosome inhibitors, such as CQ, could not further enhance the level of C protein in DENV-infected cells, though C protein exhibited high co-localization with LC3 protein, as evidenced by transmission electron microscopy and confocal assay (Wu S. Y. et al., 2021). The reason might be that the insufficient autophagic activation fails to significantly reduce the C protein level, as DENV inhibits the autophagy flux at the late stage of infection. On the contrary, DENV could also initiate autophagy flux at the early infection stage, and DENV appears to benefit from autophagy during the maturation and spread of progeny virus particles (Lee et al., 2008; Khakpoor et al., 2009; Chu et al., 2014). Moreover, DENV infection also promotes the interaction between autophagosomes and several organelles, including lipid droplets, ER, and lysosomes (Heaton and Randall, 2010; Lee et al., 2018; Zhang et al., 2018). In some cases, DENV-induced autophagy could fuse with different organelles, such as lipid droplets, and enhance viral replication (Heaton and Randall, 2010; Zhang et al., 2018). We and other groups showed that LC3 displayed a perinuclear aggregated distribution, instead of classic punctate distribution after DENV infection (Heaton and Randall, 2010; Panyasrivanit et al., 2011; Metz et al., 2015; Pu et al., 2017; Zhang et al., 2018; Kong et al., 2020). It may be due to the DENV-induced fusion between autophagosomes with different organelles or viral particles; however, this phenomenon requires further investigation. Altogether, there may be an arms race between p62 and DENV. Although p62 could mediate autophagic degradation of viral protein and restrict DENV replication, DENV also develops strategies to minimize p62 restriction and even utilizes autophagy to ensure productive infection.

Collectively, our results identified DENV C protein as a ubiquitinated substrate that could be targeted by p62 and delivered to autophagosomes for degradation with the autophagy activation. We also illustrate that p62-mediated selective autophagy contributes to the protection against DENV infection and highlight the potential anti-DENV roles of p62. Given the pivotal roles of capsid in the assembly and passage of DENV virions, our finding expands our understanding of antiviral property of selective autophagy and the development of a therapeutic intervention approach against DENV infection.

## Data Availability Statement

The original contributions presented in this study are included in the article/Supplementary Material, further inquiries can be directed to the corresponding authors.

## Author Contributions

JC initiated and designed the project. JC, JW, XG, and YW directed the research. YW, TZ, and YL performed the experiments. JH and SJ provided the technical assistance. YW, TZ, and JH analyzed the data. JC, XG, and YW wrote the manuscript. All authors contributed to the article and approved the submitted version.

## Conflict of Interest

The authors declare that the research was conducted in the absence of any commercial or financial relationships that could be construed as a potential conflict of interest.

## Publisher’s Note

All claims expressed in this article are solely those of the authors and do not necessarily represent those of their affiliated organizations, or those of the publisher, the editors and the reviewers. Any product that may be evaluated in this article, or claim that may be made by its manufacturer, is not guaranteed or endorsed by the publisher.

## References

[B1] Ames J. Yadavalli T. Suryawanshi R. Hopkins J. Agelidis A. Patil C. (2021). OPTN is a host intrinsic restriction factor against neuroinvasive HSV-1 infection. *Nat. Commun.* 12:5401. 10.1038/s41467-021-25642-z 34518549 PMC8437952

[B2] Berryman S. Brooks E. Burman A. Hawes P. Roberts R. Netherton C. (2012). Foot-and-mouth disease virus induces autophagosomes during cell entry via a class III phosphatidylinositol 3-kinase-independent pathway. *J. Virol.* 86 12940–12953. 10.1128/JVI.00846-12 22993157 PMC3497631

[B3] Bhatt S. Gething P. W. Brady O. J. Messina J. P. Farlow A. W. Moyes C. L. (2013). The global distribution and burden of dengue. *Nature* 496 504–507. 10.1038/nature12060 23563266 PMC3651993

[B4] Byk L. A. Gamarnik A. V. (2016). Properties and Functions of the Dengue Virus Capsid Protein. *Annu. Rev. Virol.* 3 263–281. 10.1146/annurev-virology-110615-042334 27501261 PMC5417333

[B5] Byk L. A. Iglesias N. G. De Maio F. A. Gebhard L. G. Rossi M. Gamarnik A. V. (2016). Dengue Virus Genome Uncoating Requires Ubiquitination. *mBio* 7 e804–e816. 10.1128/mBio.00804-16 27353759 PMC4937216

[B6] Chen M. Meng Q. Qin Y. Liang P. Tan P. He L. (2016). TRIM14 Inhibits cGAS Degradation Mediated by Selective Autophagy Receptor p62 to Promote Innate Immune Responses. *Mol. Cell* 64 105–119. 10.1016/j.molcel.2016.08.025 27666593

[B7] Choi Y. Bowman J. W. Jung J. U. (2018). Autophagy during viral infection - a double-edged sword. *Nat. Rev. Microbiol.* 16 341–354. 10.1038/s41579-018-0003-6 29556036 PMC6907743

[B8] Chu L. W. Huang Y. L. Lee J. H. Huang L. Y. Chen W. J. Lin Y. H. (2014). Single-virus tracking approach to reveal the interaction of Dengue virus with autophagy during the early stage of infection. *J. Biomed. Opt.* 19:011018. 10.1117/1.Jbo.19.1.011018 24192777

[B9] Clarke A. J. Simon A. K. (2019). Autophagy in the renewal, differentiation and homeostasis of immune cells. *Nat. Rev. Immunol.* 19 170–183. 10.1038/s41577-018-0095-2 30531943

[B10] Deretic V. Levine B. (2009). Autophagy, immunity, and microbial adaptations. *Cell Host Microbe* 5 527–549. 10.1016/j.chom.2009.05.016 19527881 PMC2720763

[B11] Deretic V. Saitoh T. Akira S. (2013). Autophagy in infection, inflammation and immunity. *Nat. Rev. Immunol.* 13 722–737. 10.1038/nri3532 24064518 PMC5340150

[B12] Dikic I. Elazar Z. (2018). Mechanism and medical implications of mammalian autophagy. *Nat. Rev. Mol. Cell Biol.* 19 349–364. 10.1038/s41580-018-0003-4 29618831

[B13] Du Y. Duan T. Feng Y. Liu Q. Lin M. Cui J. (2018). LRRC25 inhibits type I IFN signaling by targeting ISG15-associated RIG-I for autophagic degradation. *EMBO J.* 37 351–366. 10.15252/embj.201796781 29288164 PMC5793803

[B14] Feng Y. Duan T. Du Y. Jin S. Wang M. Cui J. (2017). LRRC25 Functions as an Inhibitor of NF-κB Signaling Pathway by Promoting p65/RelA for Autophagic Degradation. *Sci. Rep.* 7:13448. 10.1038/s41598-017-12573-3 29044191 PMC5647368

[B15] French A. P. Mills S. Swarup R. Bennett M. J. Pridmore T. P. (2008). Colocalization of fluorescent markers in confocal microscope images of plant cells. *Nat. Protoc.* 3 619–628. 10.1038/nprot.2008.31 18388944

[B16] Gatica D. Lahiri V. Klionsky D. J. (2018). Cargo recognition and degradation by selective autophagy. *Nat. Cell Biol.* 20 233–242. 10.1038/s41556-018-0037-z 29476151 PMC6028034

[B17] Guzman M. G. Harris E. (2015). Dengue. *Lancet* 385 453–465. 10.1016/S0140-6736(14)60572-9 25230594

[B18] Guzman M. G. Gubler D. J. Izquierdo A. Martinez E. Halstead S. B. (2016). Dengue infection. *Nat. Rev. Dis. Primers* 2:16055. 10.1038/nrdp.2016.55 27534439

[B19] Heaton N. S. Randall G. (2010). Dengue virus-induced autophagy regulates lipid metabolism. *Cell Host Microb.* 8 422–432. 10.1016/j.chom.2010.10.006 21075353 PMC3026642

[B20] Jin S. Tian S. Luo M. Xie W. Liu T. Duan T. (2017). Tetherin Suppresses Type I Interferon Signaling by Targeting MAVS for NDP52-Mediated Selective Autophagic Degradation in Human Cells. *Mol. Cell* 30:e304. 10.1016/j.molcel.2017.09.005 28965816

[B21] Jin Z. Li Y. Pitti R. Lawrence D. Pham V. C. Lill J. R. (2009). Cullin3-based polyubiquitination and p62-dependent aggregation of caspase-8 mediate extrinsic apoptosis signaling. *Cell* 137 721–735. 10.1016/j.cell.2009.03.015 19427028

[B22] Judith D. Mostowy S. Bourai M. Gangneux N. Lelek M. Lucas-Hourani M. (2013). Species-specific impact of the autophagy machinery on Chikungunya virus infection. *EMBO Rep.* 14 534–544. 10.1038/embor.2013.51 23619093 PMC3674439

[B23] Khakpoor A. Panyasrivanit M. Wikan N. Smith D. R. (2009). A role for autophagolysosomes in dengue virus 3 production in HepG2 cells. *J. Gen Virol.* 90(Pt 5), 1093–1103. 10.1099/vir.0.007914-0 19264601

[B24] Kim N. Kim M. J. Sung P. S. Bae Y. C. Shin E. C. Yoo J. Y. (2016). Interferon-inducible protein SCOTIN interferes with HCV replication through the autolysosomal degradation of NS5A. *Nat. Commun.* 7:10631. 10.1038/ncomms10631 26868272 PMC4754343

[B25] Komander D. Rape M. (2012). The ubiquitin code. *Annu. Rev. Biochem.* 81 203–229. 10.1146/annurev-biochem-060310-170328 22524316

[B26] Kong W. Mao J. Yang Y. Yuan J. Chen J. Luo Y. (2020). Mechanisms of mTOR and Autophagy in Human Endothelial Cell Infected with Dengue Virus-2. *Viral Immunol.* 33 61–70. 10.1089/vim.2019.0009 31978319

[B27] Lamark T. Svenning S. Johansen T. (2017). Regulation of selective autophagy: the p62/SQSTM1 paradigm. *Essays Biochem.* 61 609–624. 10.1042/ebc20170035 29233872

[B28] Lee Y. R. Kuo S. H. Lin C. Y. Fu P. J. Lin Y. S. Yeh T. M. (2018). Dengue virus-induced ER stress is required for autophagy activation, viral replication, and pathogenesis both in vitro and in vivo. *Sci. Rep.* 8:489. 10.1038/s41598-017-18909-3 29323257 PMC5765116

[B29] Lee Y. R. Lei H. Y. Liu M. T. Wang J. R. Chen S. H. Jiang-Shieh Y. F. (2008). Autophagic machinery activated by dengue virus enhances virus replication. *Virology* 374 240–248. 10.1016/j.virol.2008.02.016 18353420 PMC7103294

[B30] Lennemann N. J. Coyne C. B. (2017). Dengue and Zika viruses subvert reticulophagy by NS2B3-mediated cleavage of FAM134B. *Autophagy* 13 322–332. 10.1080/15548627.2016.1265192 28102736 PMC5324851

[B31] Li M. Y. Naik T. S. Siu L. Y. L. Acuto O. Spooner E. Wang P. (2020). Lyn kinase regulates egress of flaviviruses in autophagosome-derived organelles. *Nat. Commun.* 11:5189. 10.1038/s41467-020-19028-w 33060596 PMC7564011

[B32] Li Y. Hu B. Ji G. Zhang Y. Xu C. Lei J. (2020). Cytoplasmic Cargo Receptor p62 Inhibits Avibirnavirus Replication by Mediating Autophagic Degradation of Viral Protein VP2. *J. Virol.* 94:e1255–e1220. 10.1128/JVI.01255-20 32967959 PMC7925189

[B33] Liu J. Wu X. Wang H. Wei J. Wu Q. Wang X. (2021). HFE inhibits type I IFNs signaling by targeting the SQSTM1-mediated MAVS autophagic degradation. *Autophagy* 17 1962–1977. 10.1080/15548627.2020.1804683 32746697 PMC8386699

[B34] Liu K. Zhang L. Zhao Q. Zhao Z. Zhi F. Qin Y. (2018). SKP2 attenuates NF-κB signaling by mediating IKKβ degradation through autophagy. *J. Mol. Cell Biol.* 10 205–215. 10.1093/jmcb/mjy012 29474632

[B35] Liu T. Tang Q. Liu K. Xie W. Liu X. Wang H. (2016). TRIM11 Suppresses AIM2 Inflammasome by Degrading AIM2 via p62-Dependent Selective Autophagy. *Cell Rep.* 16 1988–2002. 10.1016/j.celrep.2016.07.019 27498865

[B36] Liu Y. Zhou T. Hu J. Jin S. Wu J. Guan X. (2022). Targeting Selective Autophagy as a Therapeutic Strategy for Viral Infectious Diseases. *Front. Microbiol.* 13:889835. 10.3389/fmicb.2022.889835 35572624 PMC9096610

[B37] Lu Z. Y. Cheng M. H. Yu C. Y. Lin Y. S. Yeh T. M. Chen C. L. (2020). Dengue Nonstructural Protein 1 Maintains Autophagy through Retarding Caspase-Mediated Cleavage of Beclin-1. *Int. J. Mol. Sci.* 21:9702. 10.3390/ijms21249702 33352639 PMC7766445

[B38] Metz P. Chiramel A. Chatel-Chaix L. Alvisi G. Bankhead P. Mora-Rodriguez R. (2015). Dengue Virus Inhibition of Autophagic Flux and Dependency of Viral Replication on Proteasomal Degradation of the Autophagy Receptor p62. *J. Virol.* 89 8026–8041. 10.1128/JVI.00787-15 26018155 PMC4505648

[B39] Moscat J. Diaz-Meco M. T. (2009). p62 at the crossroads of autophagy, apoptosis, and cancer. *Cell* 137 1001–1004. 10.1016/j.cell.2009.05.023 19524504 PMC3971861

[B40] Orvedahl A. MacPherson S. Sumpter R. Jr. Talloczy Z. Zou Z. Levine B. (2010). Autophagy protects against Sindbis virus infection of the central nervous system. *Cell Host Microb.* 7 115–127. 10.1016/j.chom.2010.01.007 20159618 PMC2860265

[B41] Orvedahl A. Sumpter R. Jr. Xiao G. Ng A. Zou Z. Tang Y. (2011). Image-based genome-wide siRNA screen identifies selective autophagy factors. *Nature* 480 113–117. 10.1038/nature10546 22020285 PMC3229641

[B42] Panyasrivanit M. Greenwood M. P. Murphy D. Isidoro C. Auewarakul P. Smith D. R. (2011). Induced autophagy reduces virus output in dengue infected monocytic cells. *Virology* 418 74–84. 10.1016/j.virol.2011.07.010 21813150

[B43] Pu J. Wu S. Xie H. Li Y. Yang Z. Wu X. (2017). miR-146a Inhibits dengue-virus-induced autophagy by targeting TRAF6. *Arch. Virol.* 162 3645–3659. 10.1007/s00705-017-3516-9 28825144 PMC7086938

[B44] Radivojac P. Vacic V. Haynes C. Cocklin R. R. Mohan A. Heyen J. W. (2010). Identification, analysis, and prediction of protein ubiquitination sites. *Proteins* 78 365–380. 10.1002/prot.22555 19722269 PMC3006176

[B45] Ribeiro C. M. Sarrami-Forooshani R. Setiawan L. C. Zijlstra-Willems E. M. van Hamme J. L. Tigchelaar W. (2016). Receptor usage dictates HIV-1 restriction by human TRIM5alpha in dendritic cell subsets. *Nature* 540 448–452. 10.1038/nature20567 27919079

[B46] Sagnier S. Daussy C. F. Borel S. Robert-Hebmann V. Faure M. Blanchet F. P. (2015). Autophagy restricts HIV-1 infection by selectively degrading Tat in CD4+ T lymphocytes. *J. Virol.* 89 615–625. 10.1128/JVI.02174-14 25339774 PMC4301118

[B47] Saitoh T. Fujita N. Jang M. H. Uematsu S. Yang B. G. Satoh T. (2008). Loss of the autophagy protein Atg16L1 enhances endotoxin-induced IL-1beta production. *Nature* 456 264–268. 10.1038/nature07383 18849965

[B48] Sumpter R. Jr. Levine B. (2011). Selective autophagy and viruses. *Autophagy* 7 260–265. 10.4161/auto.7.3.14281 21150267 PMC3060412

[B49] Tal M. C. Sasai M. Lee H. K. Yordy B. Shadel G. S. Iwasaki A. (2009). Absence of autophagy results in reactive oxygen species-dependent amplification of RLR signaling. *Proc. Natl. Acad. Sci. U S A* 106 2770–2775. 10.1073/pnas.0807694106 19196953 PMC2650341

[B50] Tan T. Y. Fibriansah G. Lok S. M. (2020). Capsid protein is central to the birth of flavivirus particles. *PLoS Pathog.* 16:e1008542. 10.1371/journal.ppat.1008542 32463839 PMC7255599

[B51] Viret C. Duclaux-Loras R. Nancey S. Rozieres A. Faure M. (2021). Selective Autophagy Receptors in Antiviral Defense. *Trends Microbiol.* 29 798–810. 10.1016/j.tim.2021.02.006 33678557

[B52] Wu S. Y. Chen Y. L. Lee Y. R. Lin C. F. Lan S. H. Lan K. Y. (2021). The Autophagosomes Containing Dengue Virus Proteins and Full-Length Genomic RNA Are Infectious. *Viruses* 13:2034. 10.3390/v13102034 34696464 PMC8540618

[B53] Wu Y. Jin S. Liu Q. Zhang Y. Ma L. Zhao Z. (2021). Selective autophagy controls the stability of transcription factor IRF3 to balance type I interferon production and immune suppression. *Autophagy* 17 1379–1392. 10.1080/15548627.2020.1761653 32476569 PMC8205069

[B54] Wu Y. Liu Q. Zhou J. Xie W. Chen C. Wang Z. (2017). Zika virus evades interferon-mediated antiviral response through the co-operation of multiple nonstructural proteins in vitro. *Cell Discov.* 3:17006. 10.1038/celldisc.2017.6 28373913 PMC5359216

[B55] Xian H. Yang S. Jin S. Zhang Y. Cui J. (2020). LRRC59 modulates type I interferon signaling by restraining the SQSTM1/p62-mediated autophagic degradation of pattern recognition receptor DDX58/RIG-I. *Autophagy* 16 408–418. 10.1080/15548627.2019.1615303 31068071 PMC6999607

[B56] Xiao Y. Cai W. (2020). Autophagy and Viral Infection. *Adv. Exp. Med. Biol.* 1207 425–432. 10.1007/978-981-15-4272-5_30 32671765

[B57] Xie W. Jin S. Zhang C. Yang S. Wu Y. Zhao Y. (2022a). Selective autophagy controls the stability of TBK1 via NEDD4 to balance host defense. *Cell Death Differ.* 29 40–53. 10.1038/s41418-021-00833-9 34257412 PMC8738727

[B58] Xie W. Tian S. Yang J. Cai S. Jin S. Zhou T. (2022b). OTUD7B deubiquitinates SQSTM1/p62 and promotes IRF3 degradation to regulate antiviral immunity. *Autophagy* 1–15. [Epub ahead of print]. 10.1080/15548627.2022.2026098 35100065 PMC9542415

[B59] Zhang J. Lan Y. Li M. Y. Lamers M. M. Fusade-Boyer M. Klemm E. (2018). Flaviviruses Exploit the Lipid Droplet Protein AUP1 to Trigger Lipophagy and Drive Virus Production. *Cell Host Microbe* 23:819.e–831.e. 10.1016/j.chom.2018.05.005 29902443

[B60] Zhong Z. Umemura A. Sanchez-Lopez E. Liang S. Shalapour S. Wong J. (2016). NF-κB Restricts Inflammasome Activation via Elimination of Damaged Mitochondria. *Cell* 164 896–910. 10.1016/j.cell.2015.12.057 26919428 PMC4769378

