# Supplementary material for: Autophagy Activation Induces p62-Dependent Autophagic Degradation of Dengue Virus Capsid Protein During Infection
Source: Front Microbiol. 2022 Jul 5;13:889693. doi: 10.3389/fmicb.2022.889693 (PMC9294600; doi:10.3389/fmicb.2022.889693)
Supplement: Supplementary file 1 [file Data_Sheet_1.doc]

**Supplementary Figures**

**
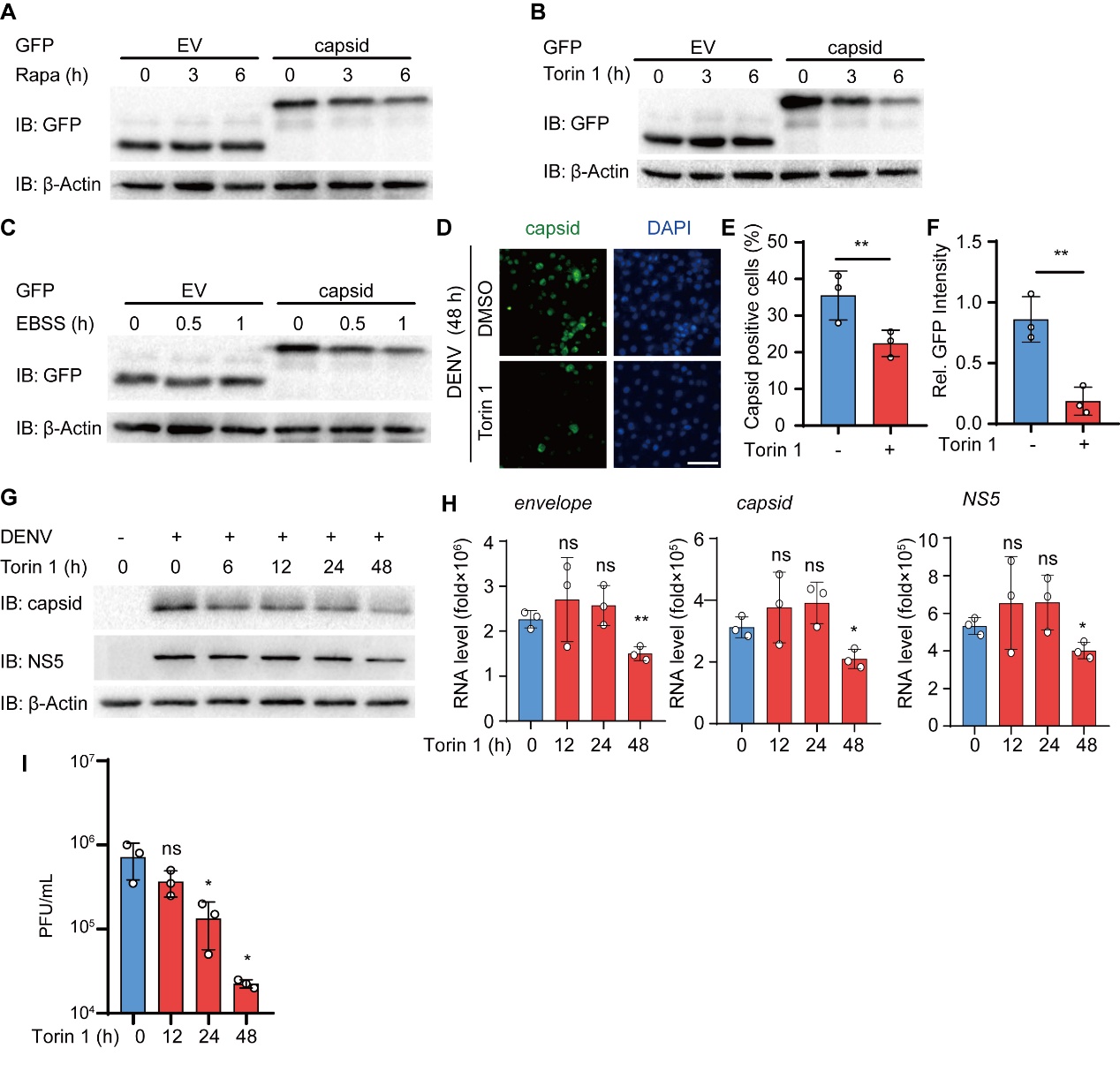
**

**Figure S1|** Autophagy inducers reduce the protein abundances of DENV capsid protein. **(A-C)** 293T cells were transfected with plasmid expressing GFP-tag C protein for 24 h, followed by 4 μM Rapa **(A)**, 2 μM Torin 1 **(B)** or EBSS **(C)** treatments for indicated time points. Cell lysates were subjected to immunoblot analysis. **(D)** Confocal microscopy of DENV (MOI=0.5)-infected Huh7 cells treated with 2 μM Torin 1, followed by labeling C protein with specific primary antibody and an Alexa Fluor 488 conjugated anti-rabbit-IgG secondary antibody (green). Scale bars, 100 μm. **(E, F)** The graphs display the relative positive cells percentage **(E)** and average fluorescent intensity in **(F)**. Data represented replicated measurements in at least 1000 cells for one independent experiment and these experiments have been replicated three times. **(G)** Huh7 cells were infected with DENV (MOI = 0.5) for 48 h followed by Torin 1 treatments at different time points, and the lysates were immunoblotted with indicated antibodies. **(H)** Huh7 cells were infected with DENV (MOI = 0.5) for 48 h followed by Torin 1 treatments at different time points. Relative RNA level of DENV *envelope*, *capsid* and *NS5* was measured by real-time PCR. **(I)** Viral plaques analysis of Huh7 cells infected with DENV (MOI = 0.5) for 48 h followed by Torin 1 treatments at different time points. Data in (**E**, **F, H & I**) are expressed as means ± SEM of three independent experiments. *p< 0.05, **p < 0.01, NS, not significant (two-tailed Student’s t-test). All the experiments are representatives of three independent biological experiments with similar results.


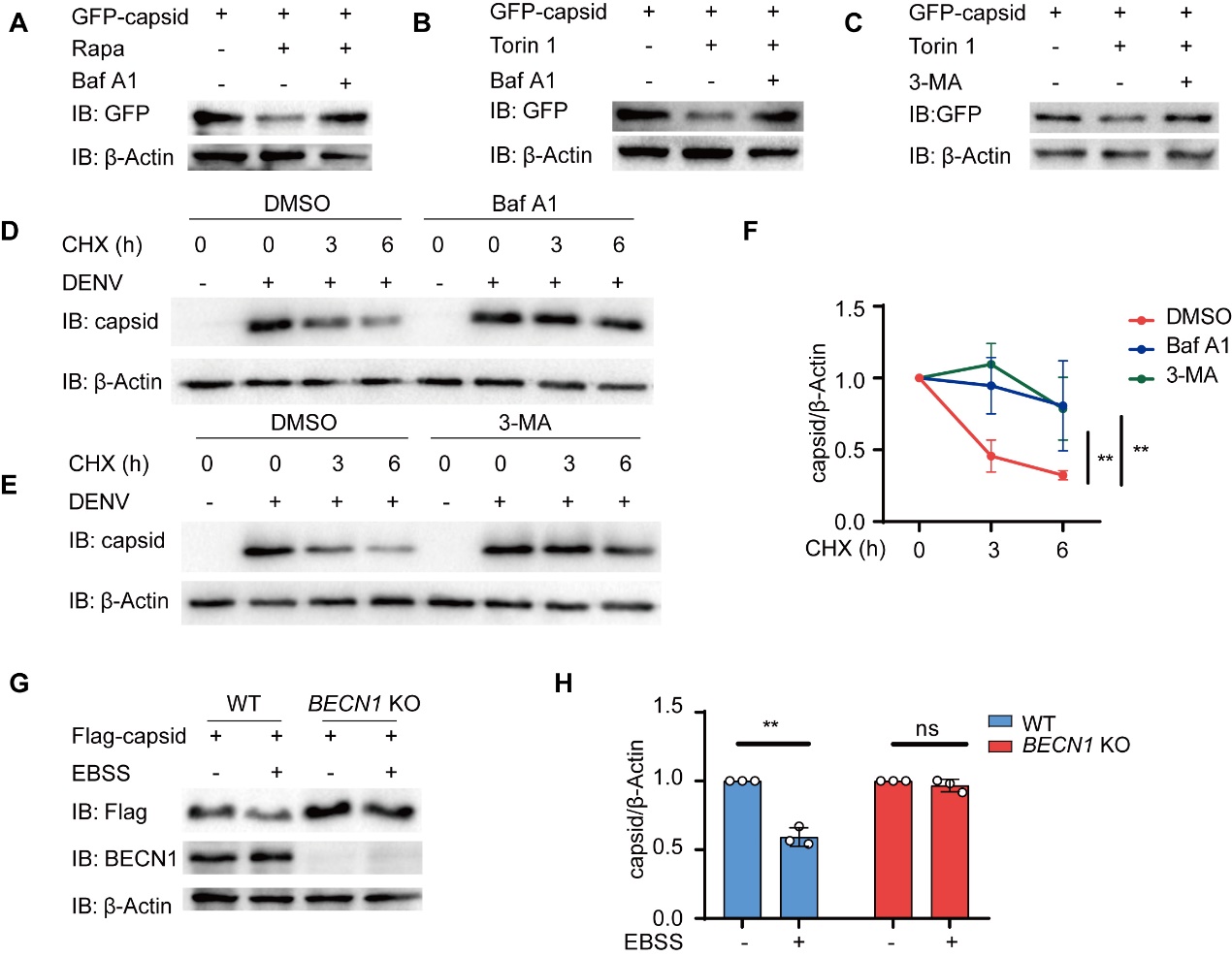


**Figure S2|** DENV capsid protein is degraded in an autophagy-dependent manner. **(A, B)** 293T cells were transfected with plasmid expressing GFP-C protein for 24 h and pre-treated with 0.2 μM Baf A1 for 6 h, followed by treatments of 4 μM Rapa **(A)** or 2 μM Torin 1 for 6 h **(B)**. The cell lysates were immunoblotted with indicated antibodies. **(C)** 293T cells were transfected with plasmid expressing GFP-C protein for 24 h and pre-treated with 10 mM 3-MA for 6 h, followed by 2 μM Torin 1 treatments for 6 h before harvested. The cell lysates were immunoblotted with indicated antibodies. **(D, E)** DENV (MOI=0.5)-infected Huh7 cells were treated with 0.2 μM Baf A1 for 6 h **(D)** or 10 mM 3-MA for 6 h **(E)**, followed by treatments of CHX (100 ng/ml) for indicated time points. Cell lysate was subjected to immunoblot analysis with the indicated antibodies. **(F)** Quantification of the protein levels of C protein from **(D, E)** were normalized to β-Actin**. (G)** Flag-C protein was transfected into WT and *BECN1* KO cells, followed by EBSS treatments for 1 h. Cell lysates were immunoblotted with indicated antibodies. **(H)** Quantification of the protein levels of C protein from **(G)** were normalized to β-Actin**.** Data in (**F**, **H**) are expressed as means ± SEM of three independent experiments. **p < 0.01, NS, not significant (two-tailed Student’s t-test). All the experiments are representatives of three independent biological experiments with similar results.

**
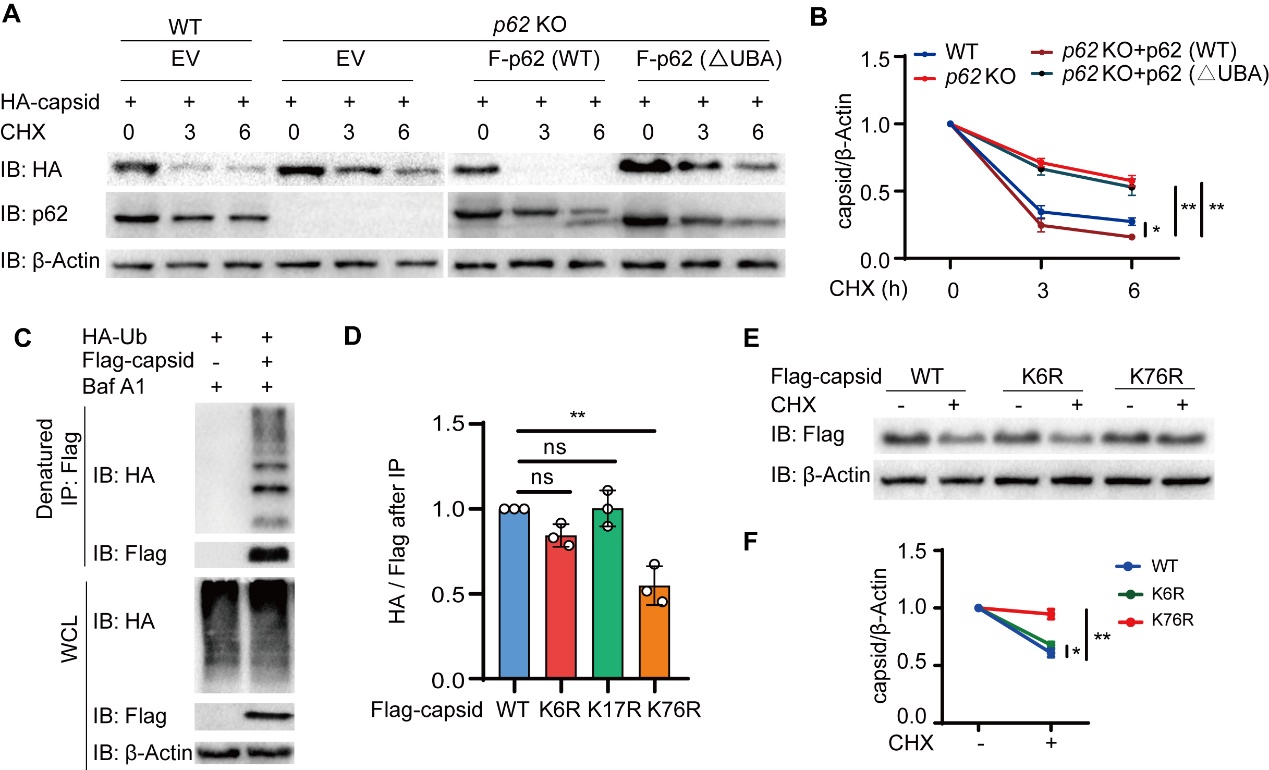
**

**Figure S3|** p62 senses ubiquitinated capsid protein. **(A)** Plasmids expressing Flag-p62 or its △UBA mutant were transfected into WT or *p62* KO 293T cells, followed by HA-C transfection for 24 h and CHX treatments for indicated time points before harvested. Cell lysates were immunoblotted with indicated antibodies. **(B)** Quantification of the protein levels of C protein from **(A)** were normalized to β-Actin. **(C)** Denatured immunoprecipitation and immunoblot analysis of 293 T cells transfected with plasmids expressing Flag-C protein and HA-ubiquitin with 0.2 μM Baf A1 treatments for 6 h. **(D)** The ratios between HA and Flag after immunoprecipitation from **Figure 5G** were analyzed. **(E)** Plasmids expressing Flag-C or its mutants (K6R or K76R) were transfected into 293T cells, followed by CHX treatments for 3 h. Cell lysates were immunoblotted with indicated antibodies **(F)** Quantification of the protein levels of C protein in **(E)** were normalized to β-Actin. Data in (**B, D & F**) are expressed as means ± SEM of three independent experiments. *p < 0.05, **p < 0.01, NS, not significant (two-tailed Student’s t-test). All the experiments are representatives of three independent biological experiments with similar results.

**
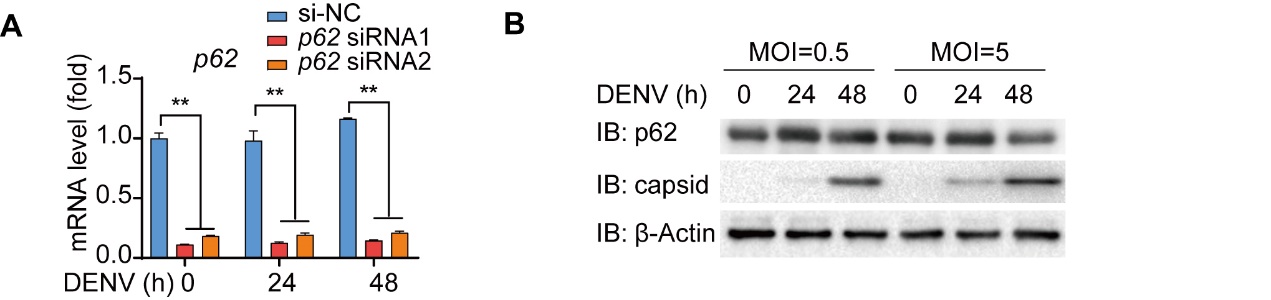
**

**Figure S4|** DENV reduces the abundance of p62. **(A)** Huh7 cells transfected with control (NC) or *p62*-specific siRNA were infected with DENV (MOI = 0.5) at different time points. Relative RNA level of *p62* was measured by real-time PCR. **(B)** Huh7 were infected with DENV (MOI = 0.5 or MOI=5) at different time points. Cell lysates were immunoblotted with indicated antibodies. Data in (**A**) are expressed as means ± SEM of three independent experiments. **p < 0.01, NS, not significant (two-tailed Student’s t-test). All the experiments are representatives of three independent biological experiments with similar results.
